# Self-Sensing of Position-Related Loads in Continuous Carbon Fibers-Embedded 3D-Printed Polymer Structures Using Electrical Resistance Measurement

**DOI:** 10.3390/s18040994

**Published:** 2018-03-27

**Authors:** Congcong Luan, Xinhua Yao, Hongyao Shen, Jianzhong Fu

**Affiliations:** 1 State Key Laboratory of Fluid Power and Mechatronic Systems, School of Mechanical Engineering, Zhejiang University, Hangzhou 310027, China; lccshdg@126.com (C.L.); shenhongyao@zju.edu.cn (H.S.); fjz@zju.edu.cn (J.F.); 2 Key Laboratory of 3D Printing Process and Equipment of Zhejiang Province, School of Mechanical Engineering, Zhejiang University, Hangzhou 310027, China

**Keywords:** carbon fibers, self-sensing, electrical resistance, load, polymers, 3D printing

## Abstract

Condition monitoring in polymer composites and structures based on continuous carbon fibers show overwhelming advantages over other potentially competitive sensing technologies in long-gauge measurements due to their great electromechanical behavior and excellent reinforcement property. Although carbon fibers have been developed as strain- or stress-sensing agents in composite structures through electrical resistance measurements, the electromechanical behavior under flexural loads in terms of different loading positions still lacks adequate research, which is the most common situation in practical applications. This study establishes the relationship between the fractional change in electrical resistance of carbon fibers and the external loads at different loading positions along the fibers’ longitudinal direction. An approach for real-time monitoring of flexural loads at different loading positions was presented simultaneously based on this relationship. The effectiveness and feasibility of the approach were verified by experiments on carbon fiber-embedded three-dimensional (3D) printed thermoplastic polymer beam. The error in using the provided approach to monitor the external loads at different loading positions was less than 1.28%. The study fully taps the potential of continuous carbon fibers as long-gauge sensory agents and reinforcement in the 3D-printed polymer structures.

## 1. Introduction

The monitoring of real-time conditions, including the sensing of strain or stress within a structure and the sensing of damage, plays a pivotal role in securing structural and operational safety or issuing early warnings on damage and deterioration. A number of sensors, such as piezoelectric, thermoelectric, electromagnetic, optical fiber, acoustic, and others, have been developed over the years to assist with structural condition monitoring [1,2,3]. Most of them can be classified as either embedded sensors or attached sensors. Nevertheless, both of them are limited in some respects: the embedded sensors are intrusive and tend to degrade the mechanical properties of the structures, while the attached sensors are less intrusive but tend to suffer from poor durability [4,5]. Furthermore, multiple sensors are required for large-scale structures because the above embedded or attached sensors can only monitor the structural conditions in the vicinity of the sensors installation.

Continuous carbon fibers are particularly attractive due to its electromechanical behavior as well as excellent mechanical properties, in which the former makes it as a potential long-gauge sensing agent and the latter makes it as a dominant reinforcement in advanced polymer composites [6,7]. Polymer composites possess self-sensing functions when embedded with continuous carbon fibers, which allows the monitoring of their own conditions without the need for embedded or attached sensors. Pioneering investigations on the piezoresistance properties of carbon fibers in terms of mechanical behavior under tensile loading have been conducted previously by several researchers [8,9,10,11], which lay the foundation for later studies that developed continuous carbon fibers as long-gauge sensing agents. Generally, the electrical resistance of continuous carbon fibers increases or decreases reversibly upon tensile or compressive strain, but irreversibly upon damage. This feature is believed to be ideally suited to monitor the real-time conditions of large-scale composites and structures.

The self-sensing functions of carbon fibers have been drawing increasing attention in recent decades. Many great efforts in the research into the electromechanical behavior of carbon fiber polymer–matrix composites under both tensile and flexural loading have been made to demonstrate the potential applications in strain or stress sensing, health monitoring, and damage detection. In the earliest studies, carbon fibers were developed as sensory agents for strain or stress sensing in composites and structures [12,13,14]. Huang and Wu [15] studied the electrical sensing properties of carbon fiber-reinforced plastics (CFRP) strips for measuring low strain levels. However, the piezoresistivity was relatively small and instable. A pre-tensioning approach and signal processing method have been provided in their follow-up research to overcome the above issues [16,17]. Wang and Chung [18] investigated the self-monitoring of dynamic strain in a continuous cross-ply [0/90] carbon fiber polymer–matrix composite through electrical resistance measurement. The electrical resistance in the longitudinal direction first decreased and then increased, while the resistance perpendicular to the fiber layers increased monotonically during static tension to failure. Angelidis et al. [19] attributed the above phenomenon to the direction of current flow influence, a positive piezoresistivity with a gauge factor of 1.75 when strained parallel to carbon fibers and a gauge factor of 2.7 when uniform current flow perpendicular to the fibers have been reported in their research. Bashmal et al. [20] compared the electromechanical behavior of continuous carbon fibers with different number of filaments for strain measurements in terms of experimental and computational methods. Wang and Chung [21] also investigated the self-sensing ability of continuous carbon fiber polymer–matrix composites under flexure. The surface resistance on the compression side decreases upon flexure, while the surface resistance on the tension side increases upon flexure.

Carbon fibers can also serve as sensing agents for effective damage detection in composites and structures [22,23,24,25,26,27,28,29]. Abry et al. [25] investigated the possibility of in situ detection of damage in unidirectional CFRP by means of electric resistance measurements. Strong changes in the resistance were measured during the development of damage owing to the modifications of the conduction paths. Kaddour et al. [30] presented an electrical technique for detecting failure in balanced angle-ply CFRP under high strain-rate tensile testing. Kalashnyk et al. [31] reported the electromechanical properties of carbon fiber bundles and strands, which indicated that both carbon fiber composites provided highly suitable and precise tests for estimating in situ fiber and composite damage. Todoroki and Omagari [32] provided a multiple electrodes method to detect the matrix crack density in CFRP laminates through the changes in slope of electrical resistance. Eight electrodes were mounted on a single surface of a specimen using silver paste, where the two outermost electrodes applied electrical current and the inner electrodes measured electric voltage changes. Kovalves et al. [33] also presented a multiple electrodes method for the detection of the presence of delamination and the localization of delamination in carbon-fiber-reinforced composites.

Several theoretical works have also been reported. An analytical model that considered the degree of current penetration for the piezoresistive phenomenon under flexure has been provided by Zhu and Chung [34]. A lamination theory to predict the piezoresistance behavior of laminate composites based on the classical thin laminate theory and electrostatic theory was provided by Xiao et al. [35]. Multidirectional loading relations between the changes in electrical resistance and the measured strains based on the theory of anisotropic piezoresistivity for single-ply carbon fiber-reinforced plastics have been presented by Todoroki et al. [36]. All these theoretical works are helpful for fundamentally understanding of the electromechanical behavior in carbon-fiber-based composites.

Although various studies have applied continuous carbon fibers as sensing agents for condition monitoring, the sensing behavior of continuous carbon fibers in terms of different loading positions still lacks adequate research. Most reported studies on the electromechanical behavior of carbon fiber composites under flexure are three- or four-point bending tests, where the load is applied at the geometric center of the structures. However, in practical monitoring applications, the external loading is likely to be applied in any position of the structure, rather than at the geometric center. It is also well-known that the load at different loading positions can lead to different stress on the same structure, which means that the capability of load bearing is closely related to loading positions. In this case, it is meaningful to monitor the loads at different loading positions because it is important for the external loading history to be recorded prior to the occurrence of damage and deterioration.

This article is devoted to develop continuous carbon fibers as long-gauge sensing agents in three-dimensional (3D)-printed polymer structures to monitor the flexural loads at different loading positions. The feasibility of continuous carbon fibers serves as sensory agent and reinforcement within 3D-printed structures have been investigated in our previous studies [37,38]. In this paper, we only focus on the self-sensing behavior of continuous carbon fibers under flexural load at different loading positions. A double-nozzle 3D printer was developed to produce the specimens, which can help to ensure that all specimens are highly consistent without affected by the manual factors. The relationship between the fractional change in electrical resistance and the flexural load in terms of different loading positions was established. Subsequently, an approach for the real-time monitoring of the external load at different loading positions using electrical resistance measurement was presented. Finally, experimental verification of the approach was conducted on a 3D-printed thermoplastic simply supported beam. The study extends the potential of the continuous carbon fiber as a long-gauge sensory agent and broadens its application for monitoring external load at different positions along the fibers’ longitudinal direction.

## 2. Theoretical Derivation and Methods

The principle of self-sensing in carbon-fiber-embedded 3D-printed polymer beams draws on the piezoresistive change in the electrical resistance of the continuous carbon fibers in response to strain:

(1) $$\frac{\Delta R}{R_{0}}=k\varepsilon =k\frac{\Delta l}{l}=\frac{k}{l}\int_{0}^{l}\varepsilon \left(x\right)dx,$$

where Δ*R* is the change in resistance, *R*_0_ is the initial resistance, *k* is the strain sensitivity, Δ*l* is the change in length, *l* is the initial length, and $\int_{0}^{l}\varepsilon \left(x\right)dx$ is the integral form of Δ*l*, where ε(*x*) designates the distribution of the strain along the fiber, which depends on the boundary conditions and the loading scheme.

In contrast to studies conducted in the past, the current study aims to investigate the self-sensing characteristics of continuous carbon fibers under flexural load at different loading positions along the fiber longitudinal direction, and to establish the relationship between the fractional change in resistance of the carbon fibers and the flexural load at different loading positions. Strain and stress are proportional to one another in the elastic regime, wherein the stress is produced by the external load. Thus, the relationship between the fractional change in resistance of the carbon fibers and the external load at different loading positions can be established based on Equation (1).

**Figure 1:**
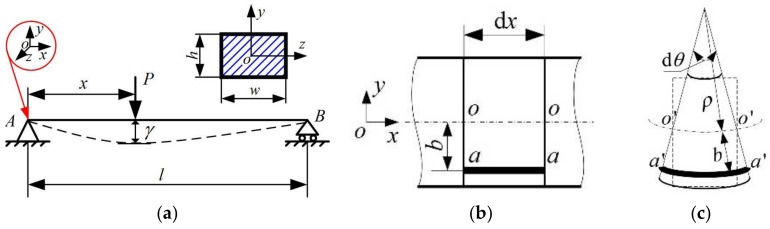
A simply supported beam model: (**a**) the loading scheme and related parameters, (**b**) the microsegment of the beam before deformation, and (**c**) the microsegment of the beam after deformation. (Note: the neutral axis of $\bar{oo}$ and $\bar{o^{\prime }o^{\prime }}$ does not coincide with the geometric center of the specimen due to the large difference in extensional stiffness of carbon fiber tow and the polymer matrix).

To establish the relationship between the fractional change in electrical resistance and the flexural load in terms of different loading positions, two assumptions based on the Euler–Bernoulli hypothesis [39,40] are cited. One is the plane hypothesis, and the other is the deformation continuity hypothesis. A simply supported beam model (as shown in Figure 1) is used to derive this relationship in terms of geometrical–physical–mechanical–electrical constitutive law, where *P* is the external load, *x* is the loading position along the fibers longitudinal direction, *b* is the distance of the carbon fiber layer from the neutral layer, *l* is the span length, *w* is the width, *h* is the height of the beam, d*x* is a microsegment of the beam, $\bar{aa}$ is a microsegment of carbon fibers, and $\bar{oo}$ is a microsegment of the neutral layer.

The position of the neutral layer can be determined according to the formula for neutral axis:

(2) $$\sum_{i=1}^{n}E_{i}S_{i}=0,$$

where *E_i_, S_i_* are the elastic modulus and static moment with respect to the neutral axis of *i*th material, respectively.

According to the Euler–Bernoulli assumptions, the following geometrical relationships of a microsegment of beam dx exist before deformation (Figure 1b) and after deformation (Figure 1c):

(3) $$dx=L_{\bar{oo}}=S_{\bar{o^{\prime }o^{\prime }}}=\rho d\theta$$

(4) $$L_{\bar{aa}}=dx=\rho d\theta$$

(5) $$S_{\bar{a^{\prime }a^{\prime }}}=\left(\rho +y\right)d\theta$$

(6) $$\varepsilon \left(x\right)=\frac{S_{\bar{a^{\prime }a^{\prime }}}-L_{\bar{aa}}}{L_{\bar{aa}}}=\frac{\left(\rho +b\right)d\theta -\rho d\theta }{\rho d\theta }=\frac{b}{\rho },$$

where $L_{\bar{oo}}$ is the length of the neutral axis and $L_{\bar{aa}}$ is the length of carbon fibers before deformation; $S_{\bar{o^{\prime }o^{\prime }}}$ is the length of the neutral axis and $S_{\bar{a^{\prime }a^{\prime }}}$ is the length of carbon fibers after deformation; and *ρ* is the radius of curvature of the neutral axis after its deformation.

Assuming that there is no extrusion between the layers that are parallel to the neutral layer, the physical relationship satisfies Hooke’s law:

(7) $$\sigma =E\varepsilon =E\frac{b}{\rho },$$

where *E* is the elastic modulus of the beam, which can be calculated based on the rule of mixture according to Equations (8) and (9):

(8) $$E=\sum_{i=1}^{n}E_{i}V_{i}$$

(9) $$\sum_{i=1}^{n}V_{i}=1,$$

where *E* is the total number of materials, *E_i_, V_i_* are the elastic modulus and volume fraction of the *i*th material, respectively.

**Figure 2:**
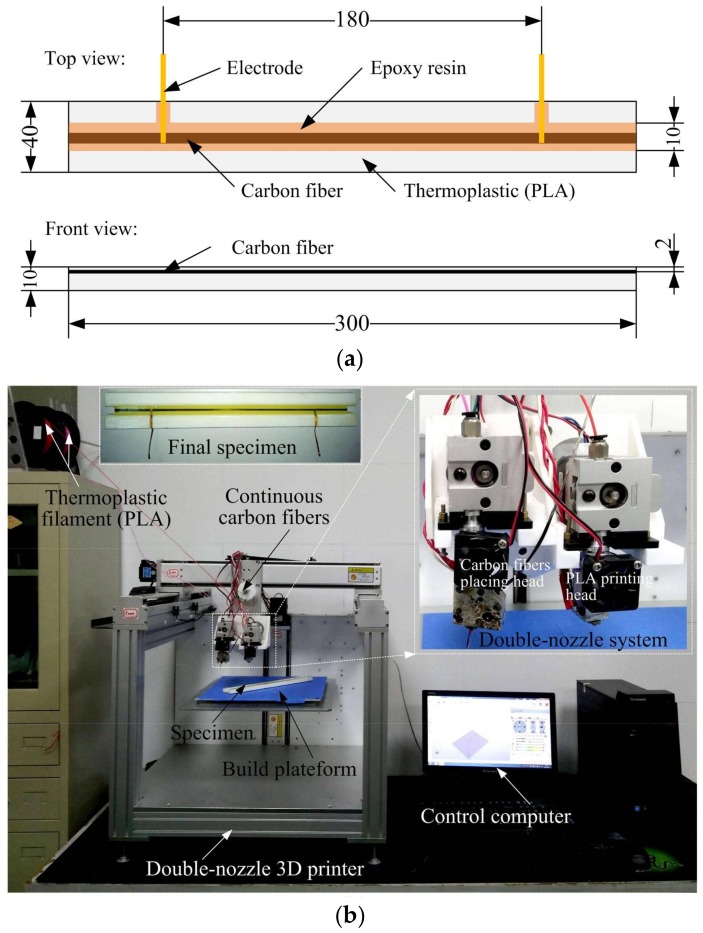
Illustration of specimen dimensions and fabrication equipment: (**a**) Dimensions of the specimen (unit: mm); (**b**) double-nozzle 3D printing system.

The normal stress σ satisfies the following three mechanical relationships:

(10) $$\begin{array}{l} N=\int_{A}\sigma dA=0\\ M_{y}=\int_{A}z\sigma dA=0\\ M_{z}=\int_{A}y\sigma dA=M \end{array},$$

where *N* is the axial force on the transverse section, *M_y_* is the moment of the couple about the *y*-axis, and *M_z_* is the moment of the couple about the *z*-axis, which can be expressed as follows in terms of Figure 2a:

(11) $$M_{z}=M_{\left(x\right)}=\left\{ \begin{array}{ll} \frac{P\left(l-x\right)}{l}x_{1} & \left(0\leq x_{1}\leq x\right)\\ \frac{Px}{l}x_{2} & \left(0\leq x_{2}\leq l-x\right) \end{array}\right..$$

Combining Equations (7) and (10), the strain on the microsegment of the carbon fiber layer can be expressed as follows:

(12) $$\varepsilon =\frac{\sigma }{E}=\frac{Mb}{E\int_{A}y^{2}dA}.$$

Combining Equations (1) and (12) gives the expression:

(13) $$\frac{\Delta R}{R_{0}}=\frac{k}{l}\int_{0}^{x}\frac{P\left(l-x\right)b}{El\int_{A}y^{2}dA}x_{1}dx_{1}+\frac{k}{l}\int_{0}^{l-x}\frac{Pxb}{El\int_{A}y^{2}dA}x_{2}dx_{2}=\frac{6kbP}{Elhw^{3}}\left(xl-x^{2}\right).$$

Equation (13) establishes the relationship between the fractional change in resistance *ΔR/R_0_* and the external load *P*, the loading position *x*, and the position *b* of the carbon fiber layer away from neutral layer. Usually, in a real project, the parameters *E*, *l*, *h*, *w*, *b*, and *k* are constant in the elastic region. Thus, the fractional change in resistance varies with the external load *P* and the loading position *x*. By introducing a factor *K_x_* (sensitivity coefficient), Equation (13) can be simplified as follows:

(14) $$\frac{\Delta R}{R_{0}}=K_{x}P,$$

where the factor *K_x_* is defined as the fractional change in electrical resistance per unit force:

(15) Kx=ΔRR0P=6kb(xl−x2)Elhw3.

The objective of using continuous carbon fibers embedded in polymer beams for the real-time monitoring of the external load at different loading positions can be achieved when the sensitivity coefficient *K_x_* is determined. Note that the sensitivity coefficient *K_x_* is different from the strain sensitivity *k*, which is related to the applied load loading positions. The strain sensitivity *k* is constant whatever external loads are applied, while the sensitivity coefficient *K_x_* should be constant only when the loading positions are determined. Experimental verifications have been conducted to validate the feasibility of the approach in the following sections.

## 3. Materials and Experiments

### 3.1. Specimen Fabrication

The specimen was designed and fabricated as shown in Figure 2. The carbon fiber was the T300B-3000-40B Torayca™ and the thermoplastic was polylactic acid (PLA). The specimen was 300 mm long, 40 mm wide, and 10 mm thick, and one carbon fiber tow (including 3000 filaments with a 7.0 μm diameter in each filament) was located 2 mm above the tension surface, as shown in Figure 2a. The specimen was fabricated using a self-developed double-nozzle 3D printer, as shown in Figure 2b. The PLA matrix was printed using the PLA printing head, and the carbon fiber tow was placed using the carbon fiber printing head after the PLA matrix printing finish. Trichloromethane was used to remove the PLA material on the carbon fibers at the positions where the electrodes were placed. Then, the copper electrodes were connected to the carbon fibers and bonded with conductive silver adhesives. Finally, an epoxy resin DY-E44 was used to stabilize the printed carbon fibers and the electrodes, along with the PLA matrix. The specimen cast with epoxy resin was cured at room temperature for 24 h. A total of three specimens were fabricated and tested.

### 3.2. Testing

**Figure 3:**
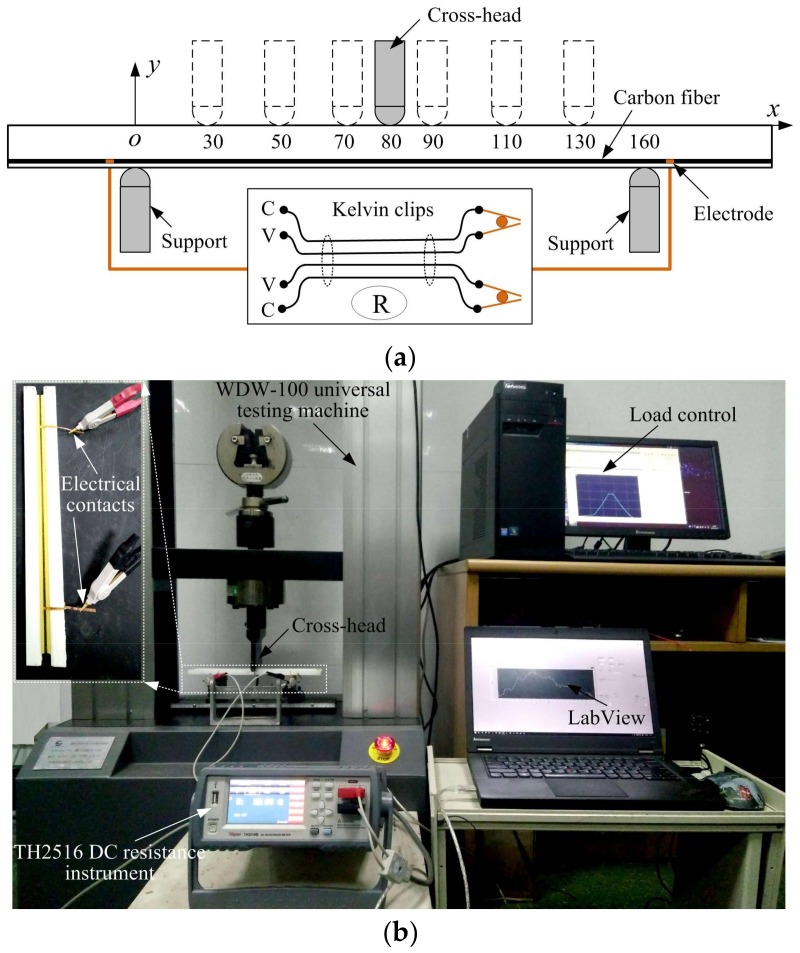
Configuration of experiments: (**a**) Schematic diagram for the measurement using Kelvin clips at different loading positions; (**b**) experimental setup for asymmetric three-point bending tests.

Figure 3 gives the schematic diagram of the measurement process of the electrical resistance at seven different loading positions. A WDW-100 microcomputer control electronic universal testing machine was used for cyclic flexure loading on the specimen at seven different loading positions. The testing machine was operated in a force control mode with a 2 mm/min loading and unloading speed. The maximum force was 110 N (about 10% of ultimate load), the minimum force was 10 N, and the force was sustained for 10 s when the force reached 110 N. The loads and the cross-head displacements were recorded during the whole loading/unloading process. The electrical resistance of the specimen was measured by a TH2516 DC resistance instrument controlled by a personal computer with the LabVIEW at the same time. All the results were obtained at a sampling frequency of 120 Hz. The experiment was repeated three times for each specimen to investigate the repeatability and reversibility of the change in electrical resistance at each loading position.

## 4. Results and Discussion

### 4.1. Response to External Load at Different Loading Positions

**Figure 4:**
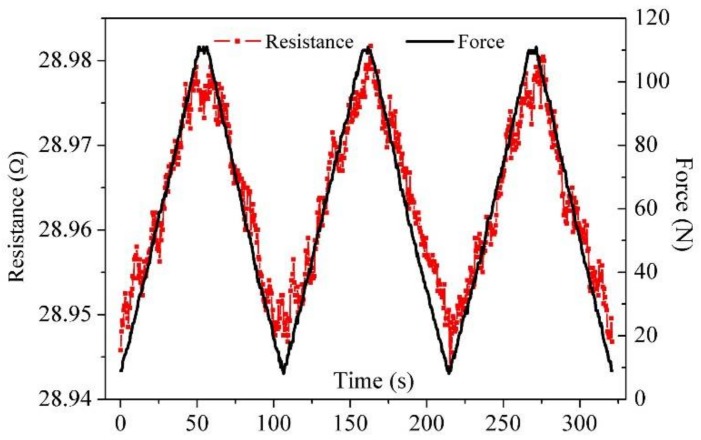
Resistance and force versus time at the loading position of 80 mm for three loading cycles.

Figure 4 shows the electrical resistance and force obtained during the cyclic loading process at a loading position of 80 mm. The electrical resistance increases linearly upon loading and decreases linearly after unloading, although accompanied by some noise. The main reason for the noise is that the contact quality between the clips and electrodes is susceptible to external disturbances during the loading process. The hysteretic effect also exists in the unloading process, and this is most obvious in the second cycle. The electrical resistance response in terms of the external load is generally consistent and repeatable, which indicates a stabilization in carbon fiber sensing agents embedded in the 3D-printed polymer structures. Compared with our previous work [36], the fluctuation component in the measured electrical resistance was significantly reduced due to the carbon fibers being placed automatically using the double-nozzle 3D printer instead of in a manual manner. The result also further demonstrates that 3D printing technology can be used to produce smart carbon-fiber-reinforced polymer structures with self-sensing functions.

**Figure 5:**
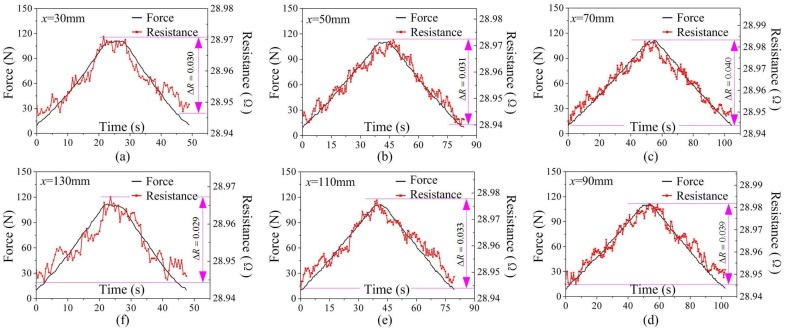
Resistance and force versus time at different loading positions in one loading cycle.

Figure 5 gives similar results for another six loading positions in the first loading cycle. It also reveals a clear correlation between the electrical resistance and the external load, but this relationship becomes weakened with the loading positions moving farther away from the central point (*x* = 80 mm). A smaller increase in electrical resistance at loading positions away from the central point due to low strain is induced by the same magnitude load at farther loading positions. The biggest increase of 0.04 Ω at the loading position *x* = 70 mm was observed, while the smallest increase of 0.029 Ω at the loading position of *x* = 130 mm. The similarity between the electrical resistance and the external force at different loading positions in the experiments indicates that the continuous carbon fiber can be used as a self-sensing agent for external load monitoring at different loading positions.

### 4.2. Determination of the Sensitivity Coefficient K_x_ Based on Experimental Results

**Figure 6:**
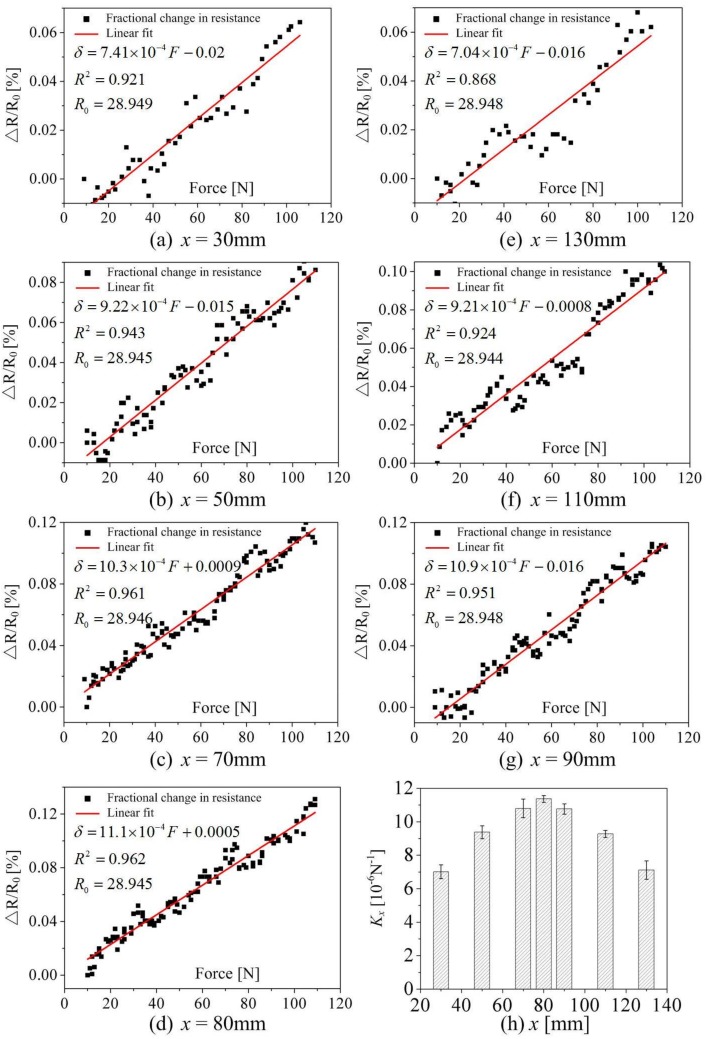
Linear fit of the fractional change in resistance versus force at different loading positions.

**Figure 7:**
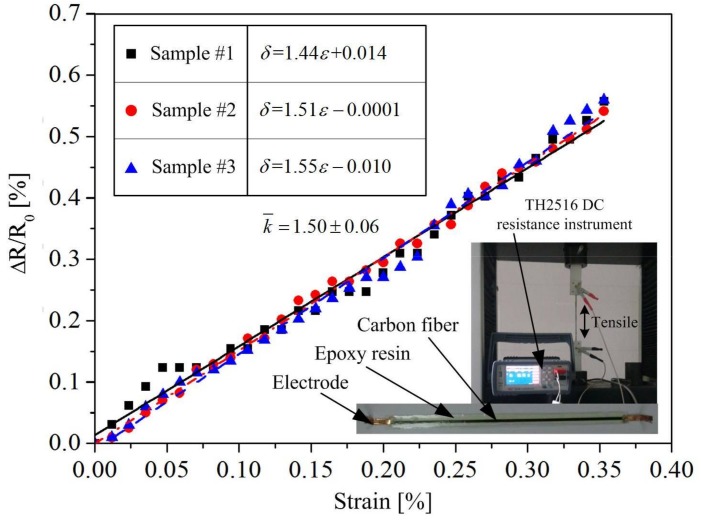
Determination of strain sensitivity k through the tensile tests.

The sensitivity coefficient *K_x_* for each loading cycle at different loading positions can be evaluated according to the linear fitting results of the detected response. Figure 6a–g gives the linear fit of the fractional change in resistance versus force at different loading positions. It can be seen that there is a strong linear relationship between the fractional change in resistance and the force at each loading position. All values of R-squared (*R*^2^), which refers to the goodness of fit and describes how well the linear model fits the set of testing data here, are >0.92, except the position *x* = 130 mm (*R^2^* = 1, which means a perfect fit). Figure 7h summarizes the average sensitivity coefficient *K_x_* and standard deviation of linear fitting results at different loading positions for three loading cycles. The average sensitivity coefficient *K_x_* was strongly dependent on the loading position, which ranged from 7.01 to 11.37 at different loading positions throughout the three loading cycles, with a maximum standard deviation of 0.56 at the loading position *x* = 70 mm and a minimum standard deviation of 0.21 at the loading position *x* = 80 mm.

Table 1 summarizes the sensitivity coefficient *K_x_* at different loading positions for the three tested specimens in the first loading cycle. It further reveals that the sensitivity coefficient *K_x_* is strongly related to the loading position, which means that the same magnitude load at different loading positions will lead to a different electrical resistance being measured. In addition, it implies that the response and correlation between the electrical resistance and external load are generally repeatable. Therefore, it is meaningful to investigate the electromechanical behavior of carbon fibers in terms of the external load at different loading positions.

### 4.3. Comparison of K_x_ between Measured and Calculated Values

To calculate the sensitivity coefficient *K_x_*_,_ the strain sensitivity *k* of carbon fiber tow used in this study was measured under the tensile test. The length of the carbon fiber tow was 180 mm, which was coated with a DY-E44 epoxy resin. The final dimensions of the specimen were 180 mm long, 10 mm width, and 2 mm thickness. The loading speed was 2 mm/min. Three samples were used during the tensile tests. The results are shown in Figure 7, and the average measured strain sensitivity *k* is 1.50, with a standard deviation of 0.06.

The sensitivity coefficient *K_x_* at different positions for the specimen adopted in this research was calculated according to Equations (2), (8), and (15). The main parameters used in calculation are listed in Table 2. The elastic modules *E_i_* of each material was provided by suppliers, and the volume fraction *V_i_* of each material was calculated according to the specimen’s dimensions.

The final calculated sensitivity coefficient *K_x_* in terms of different loading positions has the following form:

(16) $$K_{x}=1.85\times 10^{-9}\left(-x^{2}+160x\right).$$

**Figure 8:**
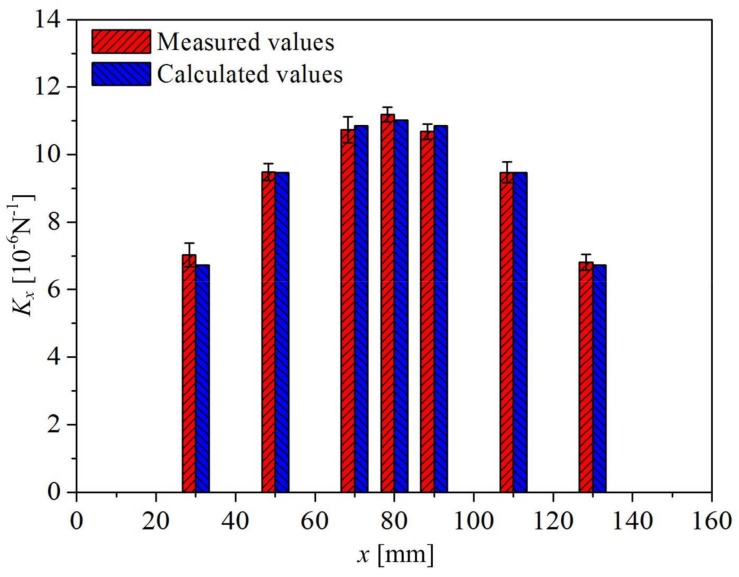
Comparison of sensitivity coefficient *K_x_* between measured values and calculated values.

Figure 8 gives out the comparison of sensitivity coefficient *K_x_* between measured values (listed in Table 1) and calculated values (according to Equation 16). It can be seen that the calculated values are mostly consistent with the measured values, which indicates the feasibility of the presented approach in this study. Thus, the external load at different loading positions on the continuous carbon-fiber-embedded polymer beam can be calculated using Equation (17), when the fractional change in electrical resistance is measured:

(17) P=ΔRR0Kx=Elhw36kb(xl−x2)⋅ΔRR0=5.41×108160x−x2⋅ΔRR0.

From Equation (15), it can be seen that the sensitivity coefficient *K_x_* is related to the polymer structure dimensional parameters (*l*, *h*, *w*, and *b*), physical parameter *E*, and the carbon fibers’ strain sensitivity, *k*. If these parameters are changed, Equations (16) and (17) would need to be recalculated.

### 4.4. Verification

**Figure 9:**
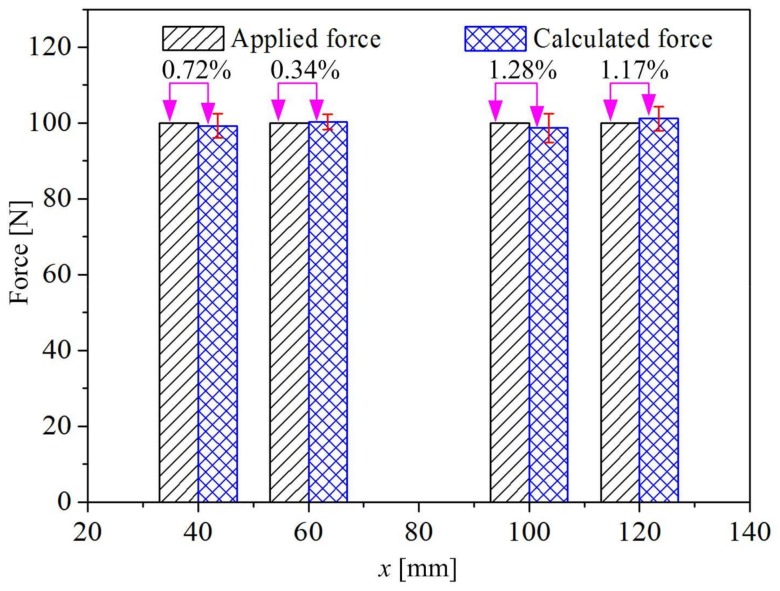
Results of monitoring loads at different positions by electrical resistance measurement.

To verify the feasibility of predicting external load based on Equation (17) using electrical resistance measurement, several loading experiments in another four loading positions (*x* = 40, 60, 100, and 120 mm) were investigated. In these experiments, the testing machine was operated in speed control mode, and the loading speed was maintained at 2 mm/min until the experimental force reached 100 N. Then, the force was controlled at 100 N for 5 s, and the TH2516 DC resistance instrument recorded the electrical resistance of the specimen with a sampling frequency of 120 Hz. The force at each loading position was calculated using the measured electrical resistance according to Equation (17). The comparison between the predicted force and experimental force is shown in Figure 9. It can be seen that the predicted results agree with the experimental results, and the maximum error is 1.28%. These results show that the approach is feasible for real-time monitoring of the external load at different loading positions using electrical resistance measurement.

## 5. Conclusions

This study explores using continuous carbon fibers embedded in 3D-printed polymer structures for self-monitoring of the external load at different loading positions. A double-nozzle 3D printer was developed to integrate continuous carbon fibers within the printed thermoplastic polymer structures, where the carbon fibers were used as a sensing element to achieve structural condition monitoring. The relationship between the fractional change in electrical resistance and the external load at different loading positions has been established theoretically and investigated experimentally, from which the following conclusions can be drawn:

(1) There has a quadratic functional relationship between the sensitivity coefficient *K_x_*, which defined as the fractional change in resistance per unit flexural load, and different loading positions. The flexural load at different loading positions can be determined through the electrical resistance measurement. Good agreement has been attained between the calculated and measured external loads, with a maximum error of 1.28%.

(2) The sensitivity coefficient *K_x_* at different loading positions can be calculated for the specific specimen when the strain sensitivity is determined. The feasibility of the proposed method has been proved by the comparison between the calculated and measured sensitivity coefficients.

(3) 3D printing technology allows the self-monitoring carbon-fiber-reinforced polymer structures to be produced in an efficient way, which also shows a great potentiality of industrial application. The 3D-printed specimens in the experiment show good repeatability and stability, with a maximum standard deviation of 0.38 in the average sensitivity coefficient among the all printed specimens.

However, further research is necessary to elucidate the following. The method provided in this paper is only suitable for the signal concentrated load prediction, and the loading position needs to be known in advance. It is powerless in the case of position-unknown loads and distributed loading or several loads. A sensing mesh composed of multiple continuous carbon fiber tows might help to achieve detection of both position-unknown loads and multiple loads, and related work will be presented in future publications.

## Figures and Tables

**Table 1:** Sensitivity coefficient *K_x_* in one loading cycle of the three tested specimens (10^−6^·N^−1^).

Loading Position	*x* = 30 mm	*x* = 50 mm	*x* = 70 mm	*x* = 80 mm	*x* = 90 mm	*x* = 110 mm	*x* = 130 mm
Specimen 1	7.41	9.22	10.30	11.10	10.90	9.21	7.04
Specimen 2	6.95	9.72	10.99	11.44	10.45	9.81	6.83
Specimen 3	6.71	9.51	10.93	11.03	10.69	9.39	6.56
Average $\bar{K_{x}}$	7.02 ± 0.36	9.48 ± 0.25	10.74 ± 0.38	11.19 ± 0.22	10.68 ± 0.23	9.47 ± 0.31	6.81 ± 0.24

**Table 2:** The main material parameters used in calculation.

Parameters	PLA Matrix	Carbon Fiber Tow	Epoxy Resin
*E_i_*	65 MPa	230 GPa	80 MPa
*V_i_*	95%	0.029%	4.971%
